# Sudden Cardiac Arrest Among Young Competitive Athletes Before and During the COVID-19 Pandemic

**DOI:** 10.1001/jamanetworkopen.2024.61327

**Published:** 2025-02-24

**Authors:** Camilla Astley, Bradley J. Petek, Randi N. Delong, Kristen L. Kucera, Barbara P. Goettsch, Kimberly G. Harmon, Jonathan A. Drezner

**Affiliations:** 1 Center of Lifestyle Medicine, Faculdade de Medicina FMUSP, Universidade de São Paulo, São Paulo, São Paulo, Brazil; 2 Department of Family Medicine, Center for Sports Cardiology, University of Washington, Seattle; 3 Sports Cardiology Program, Oregon Health & Science University, Portland; 4 National Center for Catastrophic Sports Injury Research, Department of Exercise & Sport Science, University of North Carolina at Chapel Hill, Chapel Hill

## Abstract

This cohort study compares the prevalence of sudden cardiac arrest or death among young athletes in the 3 years before vs the first 3 years of the COVID-19 pandemic.

## Introduction

Early reports during the COVID-19 pandemic raised concerns that young athletes with COVID-19 would be at increased risk of myocarditis and sudden cardiac arrest (SCA) or sudden cardiac death (SCD).^1^ Many media and social media reports insinuated that COVID-19 illness or mRNA vaccines caused an increase in SCA/SCD in athletes.^2^ Our objective was to compare the prevalence of SCA/SCD in young athletes in the 3 years before the pandemic with the first 3 years of the pandemic using data from the National Center for Catastrophic Sports Injury Research (NCCSIR).

## Methods

This prospective cohort study was approved by the institutional review board at the University of North Carolina at Chapel Hill, followed the STROBE reporting guidelines, and included a 6-year dataset from January 1, 2017, to December 31, 2022. Informed consent was obtained in survivors and next-of-kin when possible to obtain autopsy reports and medical records. The prepandemic period was defined as calendar years 2017 to 2019, and the pandemic period defined as calendar years 2020 to 2022. Cases of SCA/SCD in young athletes were identified through an ongoing surveillance program led by the NCCSIR, with detailed methods reported previously.^3^ We included competitive athletes from the youth, middle school, high school, club, college, or professional levels who experienced SCA/SCD at any time (ie, during exercise, rest, or sleep). SCA was defined as an unexpected collapse in which cardiopulmonary resuscitation and/or defibrillation was provided in an individual who survived. SCD was defined as a sudden unexpected death due to a cardiac cause or a structurally normal heart with no other explanation for death and a history consistent with cardiac-related death.^3^ For fatalities, the cause of SCD was determined by an expert panel using previously published criteria^3^ and all available information from autopsy, medical examiner, coroner, and medical records. All sources of information were used to classify race or ethnicity. If medical or postmortem records were not available or did not define race or ethnicity, then media reports and athlete photos were used to determine race or ethnicity. Race and ethnicity are included in this study because of ongoing disparities in SCA outcomes. Descriptive statistics summarized characteristics, and categorical variables were compared using the χ^2^ test, with significance defined as 2-sided *P* < .05. Data were analyzed using SAS statistical software version 9.4 (SAS Institute). Because sports participation may have declined early in the pandemic, a sensitivity analysis was performed to address uncertainty in 2020 by comparing 2017 to 2018 with 2021 to 2022.

## Results

A total of 387 SCA/SCD cases (mean [SD] age, 16.5 [2.8] years; 334 male individuals [86.3%]) were identified (Table). The observed number of SCA/SCD cases before the pandemic was not significantly different than the number of SCA/SCD cases during the pandemic (203 vs 184 cases; χ^2^_1_ = 0.93; *P* = .33). Overall survival was 50.9% (197 of 387 cases). The proportion of SCD was similar before and during the pandemic (106 of 203 cases [52.2%] vs 84 of 184 cases [45.7%]; χ^2^_1_ = 1.66; *P* = .20) (Figure). A specific cause could be determined in 139 of 190 SCD cases (73.2%) using autopsy data or coroner reports. Myocarditis was the confirmed cause of SCD in 3 cases before and 4 cases during the pandemic.

**Table.** Demographic Information for Cases of SCA or SCD During Prepandemic and Pandemic Period: January 1, 2017, to December 31, 2022

Characteristic	Patients, No. (%) (N = 387)		
	Prepandemic period (2017-2019)	Pandemic period (2020-2022)	Total
Total cases of SCA or SCD	203 (52.5)^a^^,^^b^	184 (47.5)^a^^,^^b^	387 (100.0)
Sex			
Female	27 (13.3)	26 (14.1)	53 (13.7)
Male	176 (86.7)	158 (85.9)	334 (86.3)
Age group, y			
10-12	11 (5.4)	6 (3.3)	17 (4.4)
13-15	50 (24.6)	49 (26.6)	99 (25.5)
16-18	92 (45.3)	90 (48.9)	182 (47.0)
19-21	21 (10.3)	18 (9.8)	39 (10.1)
≥22	12 (5.9)	8 (4.3)	20 (5.1)
Unknown	17 (8.4)	13 (7.1)	30 (7.7)
Race and ethnicity^c^			
African American or Black	87 (42.9)	66 (35.9)	153 (39.5)
Asian	6 (3.0)	6 (3.3)	12 (3.1)
Native American	4 (2.0)	0	4 (1.0)
White	104 (51.2)	114 (61.6)	218 (56.3)
Unknown	15 (7.4)	7 (3.8)	22 (5.7)
Athletic level			
High school	118 (58.1)	113 (61.4)	231 (59.6)
Collegiate	33 (16.3)	26 (14.1)	59 (15.2)
Youth	29 (14.3)	25 (13.4)	54 (14.0)
Middle school	15 (7.4)	14 (7.6)	29 (7.5)
Professional or semiprofessional	6 (3.0)	1 (0.5)	7 (1.8)
Other	2 (1.0)	5 (2.7)	7 (1.8)
Activity level at time of arrest			
During exercise	141 (69.5)	130 (69.5)	271 (70.0)
Within 1 h after exercise	9 (4.4)	7 (3.7)	16 (4.1)
At rest but awake	18 (8.9)	18 (9.6)	36 (9.3)
During sleep	15 (7.4)	11 (5.9)	26 (6.7)
Unknown	20 (9.9)	21 (11.2)	41 (10.6)
Fatality			
No (SCA with survival)	97 (47.8)	100 (54.3)	197 (51.0)
Yes (SCD)	106 (52.2)^d^	84 (45.7)^d^	190 (49.1)
SCD from myocarditis	3 (2.8)^e^	4 (4.8)^e^	7 (3.6)
Sporting activity at time of arrest			
Practice	55 (27.1)	58 (31.5)	113 (29.1)
Competition or game	60 (29.6)	45 (24.5)	105 (27.1)
Conditioning session	10 (4.9)	10 (5.4)	20 (5.1)
Scrimmage	3 (1.5)	3 (1.6)	6 (1.5)
Strength or weight session	3 (1.5)	1 (0.5)	4 (1.0)
Recreational activity	21 (10.3)	18 (9.8)	39 (10.1)
Nonathletic activity	42 (20.6)	34 (18.5)	75 (19.3)
Unknown	9 (4.4)	15 (8.0)	24 (6.2)

Abbreviations: SCA, sudden cardiac arrest; SCD, sudden cardiac death.

^a^ There was no statistically significant difference in the total number of cases prepandemic vs during the pandemic (χ^2^_1_ = 0.93; *P* = .33).

^b^ Sensitivity analysis excluding data from 2019 to 2020 to account for potential variations in reporting and athlete participation during the pandemic showed no statistically significant difference in the total number of SCA or SCD cases (χ^2^_1_ = 1.87; *P* = .17).

^c^ More than 1 race could be selected.

^d^ There was no statistically significant difference in the proportion of SCD prepandemic vs during the pandemic (χ^2^_1_ = 1.66; *P* = .20).

^e^ From 2017 to 2019, 2 cases of myocarditis were not tested for COVID-19, and 1 case tested positive for enterovirus on postmortem cardiac biopsy. From 2020 to 2022, 2 cases of myocarditis were not tested for COVID-19, 1 case had a negative COVID-19 nasal swab, and 1 case tested positive for enterovirus on nasal swab.

**Figure.**
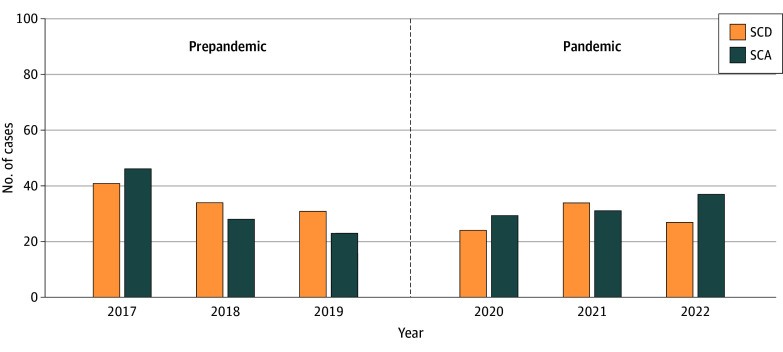
Frequency of Sudden Cardiac Arrest (SCA) and Sudden Cardiac Death (SCD) in Young Competitive Athletes in the US Before and During the COVID-19 Pandemic There were 203 cases of SCA/SCD in the prepandemic period and 184 cases in the pandemic period.

## Discussion

This cohort study found no increase in SCA/SCD in young competitive athletes in the US during the COVID-19 pandemic, suggesting that reports asserting otherwise were overestimating the cardiovascular risk of COVID-19 infection, vaccination, and myocarditis. Many athlete cases shown in social media video montages occurred before the pandemic yet claimed COVID-19 infection or vaccination raised the risk of SCA/SCD. Paratz et al^4^ also showed no association between out-of-hospital cardiac arrest and the COVID-19 vaccination in young people, and Daems et al^5^ found no evidence that COVID-19 mRNA vaccination increases the risk of SCA/SCD in athletes.

Our study is limited by the potential for missed cases, variable participation during the pandemic, including a 2.5% decline in college athlete participation in 2020 to 2021,^6^ and incomplete data on causes. Although SCA/SCD in young athletes requires more robust preventive strategies, this study suggests the COVID-19 pandemic did not increase SCA/SCD risk in athletes.

## Notes

Supplement.
Data Sharing Statement
