# Supplementary material for: Sudden Cardiac Arrest Among Young Competitive Athletes Before and During the COVID-19 Pandemic
Source: JAMA Netw Open. 2025 Feb 24;8(2):e2461327. doi: 10.1001/jamanetworkopen.2024.61327 (PMC11851236; doi:10.1001/jamanetworkopen.2024.61327)
Supplement: Supplement. — Data Sharing Statement [file jamanetwopen-e2461327-s001.pdf]

Data Sharing Statement

Astley. Sudden Cardiac Arrest Among Young Competitive Athletes Before and During the COVID-19 Pandemic. *JAMA Netw Open*. Published February 24, 2025. doi:10.1001/jamanetworkopen.2024.61327

Data
Data available: No

Additional Information
Explanation for why data not available: The data that support the findings of this study are stored and managed by the National Center for Catastrophic Sports Injury Research (NCCSIR). Restrictions apply to the availability of these data under ethics approval by the University of North Carolina (UNC) at Chapel Hill; thus, data sharing is not permissible.
